# What constitutes ‘poor’ adherence to medical advice for chronic diseases? Insights from a qualitative study among hypertension and diabetes patients in urban informal settlements, Mumbai Metropolitan Region

**DOI:** 10.1371/journal.pone.0324765

**Published:** 2025-11-18

**Authors:** Jennifer Spencer, Manjula Bahuguna, Sudha Ramani, Sweety Pathak, Sushma Shende, Shanti Pantvaidya, Vanessa D’Souza, Anuja Jayaraman

**Affiliations:** Society for Nutrition, Education and Health Action, Mumbai, India; All India Institute of Medical Sciences - Gorakhpur, INDIA

## Abstract

**Introduction:**

The problem of poor adherence to medical advice in the case of non-communicable diseases, the reasons thereof, and how these are exacerbated in low- and middle-income countries (LMICs) is well-recognized. However, there is less conceptual clarity on what ‘poor’ adherence encompasses in these settings. Conventional classifications treat poor adherence as a singular category, often disregarding its multifaceted nature. This study aimed to explore the nuances of what constitutes ‘poor’ adherence to medical advice for chronic diseases in vulnerable LMIC settings. This was done by examining the different ways in which hypertension and diabetes patients living in urban informal settlements in the Mumbai Metropolitan Region attempted to adhere to medical advice.

**Methods:**

This is a qualitative study using a grounded analysis approach. The study was part of larger research conducted to understand care-seeking for hypertension and diabetes in urban informal settlements. Purposive sampling was used to identify participants. Data was collected from September to November 2022 through in-depth interviews with 26 hypertension and diabetes patients. Emerging patterns of adherence were inductively coded and categorized using grounded analysis.

**Findings:**

The study highlights multiple ways in which patients attempted to adhere to medical advice. By tracing patient journeys and experiences in adherence, the study categorizes ‘poor’ adherence to medical advice as adherence to medication, lifestyle changes and follow-ups with various sub-categories within each. Most patients reported more than one way in which they tried to adhere to medical advice. Patients adhered well to some aspects of their medical advice and not to others, highlighting the complexities in understanding this concept.

**Conclusions:**

By understanding the nuances and complexities of ‘poor’ adherence in urban informal settlements, the study builds an empirically grounded typology on adherence. Such a typology is useful for research and practice on improving adherence to medical advice in vulnerable LMIC settings.

## 1. Introduction

Globally, it is well recognized that poor adherence to medical advice for chronic diseases can have serious health repercussions, contributing to an increase in the severity of symptoms, complications, and risk of mortality [1–3]. Tackling this issue is crucial for the success of global and national commitments to control chronic conditions [4]. Several studies have highlighted the problem of poor adherence to medical advice in the case of chronic conditions. These studies discuss multiple reasons for poor adherence, including low awareness, a lack of trust in health providers, and financial constraints arising from poverty [5–7] and emphasize how these are exacerbated in resource-constrained contexts of Low- and Middle-Income Countries (LMICs) [8,9]. However, while studies usually discuss reasons for poor adherence, the subtleties of what constitutes ‘poor’ adherence needs more clarity.

Conceptualizations of adherence have long viewed the concept as compliance of patients to treatment as prescribed by healthcare providers [3,10]. This framing has been criticized as coercive and compelling towards patients [11]. Recognizing patients’ agency in their treatments, more patient-centered definitions of adherence have emerged that emphasize concordance, where treatment decisions are collaboratively made between active and informed patients and their healthcare providers [10,11].

Based on the above definitions, poor adherence generally gets understood as deviance from compliance to treatment, or as a lack of concordance in treatment decisions between patients and healthcare providers [11]. Further, poor adherence as a concept usually gets subcategorized as being unintentional (beyond patients’ choice or control) or intentional (personal motivations/choice of patients) [10–13]. Other scholars have categorized poor adherence temporally, based on the lifecycle of treatment, differentiating between primary non-adherence (failure to initiative treatment), secondary non-adherence (failure to maintain treatment) [12,14]. Building on the above categorizations, scholars have attempted to construct quantitative scales to measure and interpret the extent of poor adherence. This includes scores and scales such as the Morisky Medication Adherence Scale MMAS-8 and Medication Adherence Report Scale MARS-5. These scales use structured surveys, most commonly measuring forgetfulness and breaks in medicines [15–18].

However, the above classifications of adherence and the measurement scales place less emphasis on the nuanced ways in which people engage with medical advice. There is limited literature that draws from the experiences of patients from vulnerable contexts in LMICs to understand what adherence encompasses as a conceptual entity in these settings. Further, the phenomenon has not been widely explored qualitatively – despite a few notable exceptions [6,19]. Existing binary categorizations of poor adherence, such as intentional versus unintentional, or primary and secondary, do not adequately capture the more complex adherence patterns of patients in LMICs, whose health-seeking journeys are convoluted [20–22].

In this context, the study qualitatively explores what constitutes ‘poor’ adherence by tracing the ways in which hypertension and diabetes patients living in urban informal settlements of the Mumbai Metropolitan Region attempt to adhere to medical advice. We chose to study hypertension and diabetes patients for understanding ‘poor’ adherence since these diseases are reported to have a high prevalence in urban India [23] with an increasing burden in urban informal settlements [17,24,25] where disease management is often suboptimal [26–29]. By providing conceptual clarity to ‘poor’ adherence in LMIC contexts, the study offers alternatives [30] to understand and categorize the concept. A grounded conceptualization of ‘poor’ adherence to medical advice from these settings can also enable us to frame contextualized intervention strategies better suited to patients’ needs in urban informal settlements.

## 2. Methods

### 2.1 Setting

The study was conducted in three urban informal settlements of a municipal corporation with a population of 0.7 million in the Mumbai Metropolitan Region, Maharashtra, India. Almost half of the population in the municipal corporation studied lives in urban informal settlements [31]. Urban informal settlements, commonly referred to as slums, are areas for residential and often commercial purposes characterized by a lack of tenure security, inadequate housing, and a congested environment with poor access to basic services like water and sanitation [32]. In the municipal corporation studied, many such settlements housed migrant populations working in nearby industries that fueled the growth of these settlements. In terms of healthcare, the study area had access to a mix of public and private providers. First-contact care was usually sought by people from private, non-allopathic doctors (with degrees or diplomas in Ayurveda, Unani, or Homeopathy) who had small clinics in these settlements. Some patients also visited private allopathic doctors. The public health system, including one secondary-level hospital and 15 primary-level facilities in the area, was not usually accessed for non-communicable disease (NCD) care.

### 2.2 Study design

The study employed qualitative methods to examine patterns of adherence to medical advice. This study was part of a larger research conducted to understand care-seeking for hypertension and diabetes in urban informal settlements in the region [20]. The larger research study employed quasi-inductive approaches to examine patient journeys. Such approaches allow context-specific theorization grounded in data from research participants, even while pragmatically allowing learnings from existing theories and frameworks to be incorporated in the analysis [33,34]. For this study, we iteratively analyzed and coded data related to adherence from the larger research study using a grounded theory analysis [35] to build context-specific theorizations of adherence. The data comprises interviews with patients diagnosed with hypertension or diabetes (or both) who shared stories on ways in which they adhered to medical advice given to them.

### 2.3 Participant selection

This study was conducted in the intervention areas of the Society for Nutrition, Education and Health Action (SNEHA), a non-governmental organization working in urban informal settlements in Mumbai since 1999. The field staff in SNEHA have strong personal ties with the community and initially identified participants based on the diversity criteria suggested by the research team. The field staff works in the area to implement various initiatives by SNEHA, for which they regularly conduct home visits. During these visits, they identified adults from the family who had hypertension and diabetes. They explained the purpose of the study to potential participants and inquired about their willingness to participate. Thereafter, researchers from SNEHA conducted the interviews and discussions at the participants’ convenience and after a formal process of informed consent. The participants were purposively sampled based on diversity in age, gender and years of diagnosis/treatment of the disease. Patients above 18 years of age with a confirmed diagnosis of hypertension, diabetes or both were included in the study.

As is the practice in qualitative studies, the exact sample size was not pre-determined [36]. Information redundancy was used as a criterion to ensure data saturation until which the recruitment of additional participants was done [37]. None of the participants approached refused participation.

### 2.4 Data collection

Data collection was done from 30^th^ September to 18^th^ November 2022. A total of 26 in-depth interviews were conducted with hypertension and diabetes patients in either Hindi or Marathi languages. Authors MB, JS, and SR, female public health professionals, conducted the interviews; all three are well-trained in qualitative data collection methods and familiar with the local context. Members from the research team at SNEHA, who were not part of the program implementation and did not regularly interact with the community, did the data collection. This helped ensure the trustworthiness of the data by limiting personal bias. The interviews were conducted in participants’ homes and lasted for 30 minutes on average.

We used an in-depth interview guide for data collection. The interview guide was developed using existing literature in the field [21,22,38]. This interview guide was used for the larger study aimed at understanding the care-seeking journeys of NCD patients within the informal settlements [20]. The interview guide was not prior pilot tested, although it was iterated and modified based on participant responses and emerging themes during the data collection. Table 1 details the key points discussed during the interviews.

**Table 1:** Key points discussed with the participants.

Nature of medical advice prescribed and followed (adherence patterns) Diverse ways in which patients tried to adhere to medical advice, e.g., medication, lifestyle changes (diet and exercise), and follow-ups (doctor visits and testing) Attitudes and practices towards adherence to medical advice Reasons for following or not following medical advice Other disease management-related information: ◦ Details of care-seeking journeys- symptoms, screening, diagnosis, complications etc. ◦ Types of healthcare providers accessed and challenges thereof ◦ Awareness and attitudes towards the diseases

### 2.5 Data analysis

Data analysis was done simultaneously with data collection [39]. After every visit, authors JS, SR, MB, and AJ, female public health professionals, conducted data debriefing sessions to discuss learnings and emerging themes. All interviews were audio-recorded and thereafter translated and transcribed simultaneously into English for analysis. Data transcription was done from October to December 2022. Authors JS, SR, MB and AJ discussed emerging ideas from the transcripts to arrive at a standard set of codes for the larger study. The transcripts were sorted and coded using NVivo Version 10.3. To ensure accuracy of the transcripts, authors JS and MB, who conducted the data collection, cross-checked the audio recording with the transcribed content and made any necessary changes. Member checking through validation of transcripts was not feasible with participants; nevertheless, during the interview, researchers ensured validity by repeating, clarifying or cross-checking narratives with participants. The themes of the findings were validated by sharing them with SNEHA’s program team, who are working in and well-versed with the community where the participants resided.

For the analysis specific to this paper, data on adherence was used from the interviews, analyzed inductively using a grounded analysis approach [35]. Grounded analysis is an inductive process where data are analyzed and coded in iterative cycles to identify patterns and themes contributing to developing or nuancing theory on a particular subject. Substantive coding [40], used in grounded analysis which includes open and axial coding, was used to frame adherence typologies. The various types of adherence, as reported by participants, were separately coded as initial (open) codes through a detailed line-by-line reading of the transcripts. These were categorized into focused or axial codes consisting of adherence typologies that club several initial codes. We further clustered the emerging patterns of adherence from the data into three broad inductive categories- adherence to prescribed medications, adherence to lifestyle change recommendations, and adherence to instructions for follow-up visits and testing. Author JS did the coding for the present study. Thereafter, the categories were refined and clustered based on discussions by authors JS, SR, MB and AJ.

An audit trail [41] was maintained throughout the study, consisting of raw data (interview transcripts, field notes), summary analysis, codebook and discussions. All discussions regarding the emerging findings, challenges and reflexivity were collectively done among authors to ensure consistency and traceability of the research.

We have referred to the Consolidated Criteria for Reporting Qualitative Research- COREQ checklist [42] in reporting our methodology. The findings have been presented using inductively analyzed categories with case stories and patient narratives under each category to illustrate the nuances of adherence in our setting.

### 2.6 Ethical considerations

Ethical approval for the research was obtained from Sigma Research and Consulting Pvt. Ltd. (Approval No. 10056/IRB/22-23). Recorded verbal informed consent was obtained from the participants. The use of verbal consent was approved by the review board, owing to COVID-19 guidelines during the data collection period. All the participants gave permission to audio-record the interviews. The consent and ensuing interactions were recorded on a smartphone device.

Participants were interviewed in places they considered convenient and safe, and with a level of privacy as indicated by them. In some cases, participants were comfortable speaking with their family; this also helped the researchers get a better perspective of the case when family members shared their experiences regarding the patient’s health-seeking journeys and adherence. The researchers paid utmost care and attention to ensure that the participants were comfortable sharing their experiences. Post-data collection, all the data transcripts were anonymized to ensure that personal identifiers such as names were excluded from data analysis and dissemination to avoid participant identification. The data was stored on SNEHA’s password-protected servers, access to which was restricted to the research team and the Director in the organization.

## 3. Findings

### 3.1 Demographic details

Eighteen out of 26 participants interviewed were above 50 years of age. An equal number of men and women participants (13 each) were interviewed. More than half (17) of the participants had resided in the area for over twenty years. Twelve participants were employed, while the rest were either unemployed or retired. Nine were diagnosed with hypertension, ten with diabetes and seven with both hypertension and diabetes. Eleven participants were diagnosed two years prior to the interaction, nine had been diagnosed between three and ten years prior to the interaction, and six were diagnosed more than ten years prior to the interaction.

### 3.2 ‘Poor’ adherence: categorizing patients’ attempts at adhering to medical advice

The study explores the nuances of ‘poor’ adherence in urban informal settlements using patient narratives on how they attempted to adhere to medical advice. Medical advice has been categorized into medication, lifestyle changes and medical follow-ups. These three categories are discussed separately below.

#### 3.2.1 Medication.

Long-term medication was a primary component of medical advice given to patients. In our setting, most participants interviewed, reported being unable to completely adhere to the prescribed medication regimens, though they tried to do so in multiple ways and to varying extents. The different ways they partially adhered to their prescriptions are described as follows.

***Making minor adjustments to medication***: Some participants reported making minor adjustments to prescribed medication without consulting their doctors. Patients either adjusted their doses or adjusted the dosage timings. Some reported skipping medicine once in a while. Patients made such minor adjustments to medicine for many reasons. One reason was forgetting to take medicine. Other reasons included forgetting to replenish medicine strips or being unable to do so on time. Case 1 elaborates on one such patient story.

**Case 1. Adjusting medicine dosage**

A 52-year-old male patient diagnosed with hypertension for eight years skipped medicines for a few days once in a while. During his initial diagnosis, he had visited several private doctors in his locality without success. He had finally been diagnosed with hypertension and operated on for bypass surgery at a public hospital 50 km away in Mumbai city. Since then, he prefers to get his hypertension medicines only from that hospital. Although he tries to take medicines regularly, there is a time gap between his medicines getting over and him getting a new stock of medicine. He doesn’t trust the local public hospital, the only place in the vicinity to get affordable medicines. Due to this, he either skips medicines for a few days or, if fewer tablets are left, he reduces the doses to make the strip last longer. – “*If there is less medicine at times, I eat accordingly or manage.*”

This case highlights how the unavailability of quality public health services in the vicinity of the urban informal settlement led patients to make minor adjustments to their medication regimes.

Patients who made such adjustments, like in Case 1, often believed that these did not adversely impact their disease prognosis or health; they looked at these adjustments as minor practical tweaks rather than deviations from prescriptions.

***Mixing medications***: In the vicinity of the urban informal settlements, a range of alternative medicine providers (with degrees or diplomas in Ayurveda, Unani, or Homeopathy) and allopathic providers had their practice. Some participants mentioned alternating or mixing medications prescribed or obtained from both allopathic and alternative medicine practitioners. Participants shared that mixing medications could provide faster relief from symptoms, enabling them to easily get back to their daily work or household activities. They also believed alternative medications had fewer side effects, preferring them for the long term. Patients considered alternative medicines to be more affordable in comparison to allopathy.

Case 2 illustrates the medication regime of one patient who routinely used Ayurvedic medicine to alleviate her symptoms and used allopathic medicine only when the symptoms became severe.

**Case 2. Mixing medications**

A 60-year-old female patient was diagnosed with hypertension and diabetes two years prior to the interaction. After diagnosis, she reported taking diabetes medicines prescribed by local doctors, which only partially alleviated her symptoms. After changing multiple doctors, she went to a trust hospital (outside the metropolitan region), whose allopathic medicines she took for a few months.

However, due to financial constraints, she stopped those medicines - *“The allopathic medicines benefit me more, but I use them only for emergency purposes.”* Instead, she buys a cheaper Ayurvedic syrup from a local Ayurvedic vendor- *“If we have money, I get the allopathic medicine. Otherwise, I take Ayurvedic medicine.”* According to her, Ayurvedic medicine is cheaper but does not provide instant relief. To alleviate her symptoms, once in a while, she purchases quick-fix allopathic medicines: *“I do have problems due to not taking (allopathic) medicine. All night, I sit until it is morning. I feel pressure in my chest and a feeling of beating hard and fast. If I feel very bad, then I go and get a sleeping pill and sleep a little peacefully.”*

Case 2 also shows how some patients resorted to quick-fix medicine to alleviate hypertension and diabetes symptoms, particularly consuming allopathic painkillers rather than or along with their prescribed medicine. Due to their daily household activities and daily wage work, patients focused on fast relief rather than comprehensive care. Low awareness regarding the symptoms of their disease often led patients to prefer quick-fix medications. For instance, one patient with hypertension told us:

“*I just told them (the staff at the public health post) that I had pain in my legs and that my body was also paining. They gave me some medicine. I didn’t tell them that I have high BP (blood pressure).*” (Female, 45 years, diagnosed with hypertension, 16 years prior to the interaction)

The nature of residence and livelihood also influenced mixed medication patterns. For instance, some residents in the settlements studied were seasonal migrants owing to the industrial/commercial job opportunities in the region, who managed their disease through a mix of medicines from their home town and their place of work:

*“My doctor is in Solapur (home town 400km away). I get two to three months of BP (blood pressure) allopathic medicine from there when I come here. If I have any problem here, I go to the local doctor (Unani). He writes me a different medicine, and I take this pill (from the allopathic doctor) in the morning and his pill (from the Unani doctor) in the evening.”* (Male, 86 years, diagnosed 12 years prior to the interaction)

***Changing medications*:** Several participants reported changing their medication for reasons other than clinical recommendations. These changes often included alternating between multiple treatment regimens prescribed by different doctors they consulted. Participants reported following these treatment regimens according to their beliefs and convenience, often because of poor doctor-patient relations and low trust in doctors. People also attempted to switch to cheaper medication and constantly explored the options available to them:

“*The medical store suggested that I could try a similar medicine from another company at half price, so I took one strip. But it did not suit me, so I went back to my old medicines.*” (Female, 57 years, diagnosed with hypertension 5 years prior to the interaction)

Patients most often explored these options on the advice of neighbors or owners/workers of pharmacies, and without formal doctor consultation.

***Breaks and stops in medication:*** Around half the participants reported taking breaks from their prescribed medications or stopping these completely. These breaks and stops often depended on patients’ perception of their disease severity. Several participants reported that they stopped medicines when they “felt better” and restarted them after symptoms resurfaced or worsened; Case 3 explains the travails of one such patient.

**Case 3. Breaks in medication**

A 62-year-old male patient diagnosed with hypertension and diabetes two years prior to the interaction, took a break in his medicines when he felt better*-* “*I did not feel anything was wrong with me, so I stopped the medicines for a month.*” Thereafter, his eyes started hurting, and when he tried to soothe them, he got a cut in his eye that did not heal for two months. Following this, he went to a few eye specialists. One eye specialist related this issue to diabetes and enquired whether he had stopped any medicines- “*He asked me about diabetes, and I said I stopped the medicines of my own will. The doctor told me never to stop this medicine, that it would affect my eyes. After this incident, I decided I was never going to stop; I have it regularly now.*”

We also encountered patients who felt compelled to stop medicines due to poor services at public health facilities. In one case, a patient’s poor experience during pregnancy in the public hospital and the rude attitude of the staff hindered her from going there for cheaper diabetes medicines:

“*It was actually a problem with money, so I have stopped (medicines) for the last one and a half months. In the public hospital, if I ask something, they will answer rudely. Whatever the problem is, we will not go to a public hospital. I will stop taking medicine for some time, but I will not go there.*” (Female, 32 years, diagnosed with diabetes six months prior to the interaction)

Further, we found that patients with comorbidities prioritized one medicine regimen over others. For instance, one patient stopped her diabetes medicine when she started treatment for tuberculosis:

“*I stopped taking diabetes medicines on my own. I was taking tuberculosis medicines, so I stopped them. Should I have tuberculosis medicines or diabetes medicines? How many can I have?*” (Female, 50 years, diagnosed with diabetes 12 years prior to the interaction)

Comorbidities made it difficult to adhere to multiple treatment regimes, eventually leading to breaks in medicine regimens that patients considered less problematic or urgent. When multiple treatments were prescribed, patients were inclined to prioritize one over the other at their discretion rather than seek medical advice.

#### 3.2.2 Lifestyle changes.

Lifestyle changes are integral to comprehensive care for chronic diseases like hypertension and diabetes. In this section, we explore how patients tried to adhere to medical advice related to diet and exercise.

***Partially following dietary advice:*** A majority of the participants reported partial adherence to diets as recommended by their healthcare providers (Refer to Case 4). The importance of diet in the comprehensive treatment of hypertension and diabetes was not always recognized by patients or shared by doctors, leading to patients partially following dietary recommendations as per their convenience and ability.

**Case 4. Partially following dietary advice**

A 35-year-old female patient was diagnosed with diabetes during her pregnancy, seven years prior to the interaction. Since her delivery, she had stopped medication and did not have any symptoms, but was later re-diagnosed with the disease, prompting her to restart medication. Her doctor advised her to follow a diet low in sugar and carbohydrates. However due to experiencing no symptoms and having a questioning attitude towards the efficacy of diet in alleviating her disease, she only partially follows dietary prescriptions: “*It doesn’t happen suddenly (stopping certain foods like potatoes and rice), but yes, I try not to eat. I don’t have the habit of eating only roti (flatbread). Unless I eat rice, I don’t feel full. So, I must eat a little bit of rice.*” She further mentioned a lack of family support due to which most of her disease management is based on as much as she can do for herself: “*They (doctors) say I should look after my diet properly, but I don’t have anybody to look after me in the house.*”

***Following diets based on local knowledge***: Few participants reported following dietary practices based on their perceptions and experiences of which diets were helpful:

*“Sometimes in the night when I feel uneasy, I need to eat sweets, say, chocolate, jaggery, biscuits, etc., so I always have it on my bedside. The doctor has not told me this, I know this as I have had high sugar for many years now.”* (Male, 75 years, diagnosed with diabetes 12 years prior to the interaction)

Such diets were not prescribed or suggested by healthcare providers but often depended on advice from neighbors and family.

***No dietary recommendations followed*:** Some participants reported that they were unable to follow diets or did not follow diets. Compulsions related to livelihood, finances, or household duties often prevented patients from adhering to diets even if they wanted to.

*“The doctor has said to eat wisely, you can’t eat a lot of things. But I am unable to do it. I cook meals for around fifteen people from my village and earn some meager amount from that. So, I have to eat whatever I cook for others.”* (Female, 45 years, diagnosed with diabetes 15 years prior to the interaction)

Similar to the above quote, patients working in factories or warehouses had meals at their workplace, where the food was not customized as per their requirements, such as low salt or no sugar. For some patients, home-cooked meals were also limited to cereals and pulses; due to financial constraints, patients could not afford to have prescribed diets of fruits, vegetables, or eggs, which were perceived as more expensive.

At times, participants shared that the doctors they consulted had not given them any dietary advice. For instance, a 62-year-old male diagnosed with hypertension and diabetes two years prior to our interaction does not follow any dietary restrictions: “*The doctor told me, eat what you want to, just don’t stop the medicine. So, I eat everything; I don’t limit eating any food*."

***No exercise recommendations followed***: Except for a few participants, most did not specifically report exercising as part of their medical advice. Exercise recommendations when received from doctors were limited to suggestions for walking and doing physical activity, without providing a specific exercise regimen. Patients in our setting performed arduous work daily, both in factories, warehouses and the household, which they considered exercise or physical activity. Doing physical activity in addition to their daily work was considered a remote possibility, as seen in Case 5.

**Case 5. Daily work as exercise**

A 40-year-old female patient diagnosed with hypertension three years prior to the interaction also suffered from thyroid disease. She mentioned that if she stops taking medicines, she has severe body aches. The doctor has asked her to exercise and take walks, but she considers this a remote possibility given her situation: “*I am a housewife. Where can I go for a walk? I wake up early in the morning, prepare breakfast, clean the house and utensils, prepare food, and do other household things. Till I complete all my work, it’s three in the afternoon. I tell him (the doctor) that the housework gives me enough exercise.*” She recalls walking earlier to nearby places, but now she feels she is unable to walk due to her disease.

Often covert in such narratives were the limitations arising from the context of urban informal settlements with dense and congested housing, inadequate access, and a lack of open spaces, which made “taking a walk” or exercising unfeasible. From the patient narratives, it appeared that most doctors did not prescribe exercise as a part of medical advice, highlighting a disconnect between lifestyle change recommendations that are otherwise an integral part of medical advice for chronic diseases, and the lived realities in our setting.

#### 3.2.3. Medical follow-ups.

In this section, we explore how patients followed medical advice related to follow-up care to monitor their disease, such as visits to the doctor after diagnosis and routine testing for blood pressure and sugar.

***Sporadic follow-ups***: A majority of the participants underwent medical follow-ups only if they felt unwell or if their symptoms worsened. Most participants believed that follow-up visits to the doctor were not necessary and that they were not financially viable. Some participants went for repeat testing directly to pathology laboratories nearby and often self-interpreted the results without showing the reports to a doctor, as seen in Case 6.

**Case 6. Sporadic follow-ups**

A 53-year-old male patient diagnosed with diabetes five years prior to the interaction tried several doctors. There were some doctors whose medicines did not suit him, and others who spoke rudely: “*Now, I don’t go to him (the doctor). I don’t go to any doctors now. I go to the lab on my own if I have to check.*” Consequently, he does his blood sugar test based on convenience, “*if I feel like it.*” He directly goes to a nearby diagnostic laboratory without consulting the doctor. He only visits his local private family doctor if his sugar levels have increased ‘alarmingly’ in the test reports from his perspective.

***No follow-ups***: Some participants shared that they did not go to the doctor for routine check-ups and/or did not do any follow-up tests to monitor their chronic conditions. Such patients continued their initially prescribed medications directly from the pharmacy.

“*If I feel any discomfort, I buy medicine from the pharmacy and take it. If I go to the doctor to check my blood pressure, he will charge me fifty rupees. Instead, it is better to take the medicine directly.*” (Female, 45 years, diagnosed with hypertension, 16 years prior to the interaction)

Patients reported financial constraints as the major reason for not visiting doctors or doing regular tests, but our discussions also revealed that patients were often unaware of the need to monitor their disease regularly.

### 3.3 One patient, multiple ways of adherence

We have elaborated in detail on the various ways patients have attempted to adhere to medical advice in our setting. It is essential to note that each patient reported more than one way in which they tried to adhere to medical advice. Patients also shifted from ‘optimal’ adherence to ‘poor’ adherence and vice versa during their patient journeys. Therefore, the ‘poor’ adherence of each patient cannot be clubbed into one type or category and instead needs to be seen as a range of patient-wise adherence patterns, as represented in Table 2. To understand the adherence patterns holistically, Table 2 also includes ‘optimal’ adherence categories, i.e., those who had reported having adhered to medical advice on medication, lifestyle changes, and follow-ups (Refer to S1 Table for details on patients who reported optimal adherence to medical advice).

**Table 2:** Patient-wise adherence patterns.

Patient Sr. No.	‘Poor’ Adherence										‘Optimal’ Adherence		
	Medication				Lifestyle Changes				Follow-ups		Medication	Diet	Follow-ups
	Minor adjustments to medication	Mixing medications	Changing medications	Breaks and stops in medication	Partially following dietary advice	Following diets based on local knowledge	No dietary recommendations followed	No exercise recommendations followed	Sporadic follow-ups	No follow-ups			
1													
2													
3													
4													
5													
6													
7													
8													
9													
10													
11													
12													
13													
14													
15													
16													
17													
18													
19													
20													
21													
22													
23													
24													
25													
26													

Table 2 presents ‘optimal’ adherence and each of the sub-categories within the three categories of ‘poor’ adherence to medication, lifestyle changes and follow-ups, followed by each participant. The table cells marked in grey represent the participants’ reported ways of attempting to adhere to medical advice, while blank cells represent the absence of those categories in a participant’s adherence journey. For instance, participant 1 optimally adhered to medication and diet while engaging in sporadic follow-ups; cells corresponding to those sub-categories are accordingly marked in grey.

Table 2 shows that most patients adhered to medical advice partially. Various ways of changing medications and breaks and stops in medication were the most common types of ‘poor’ medication adherence. Partially following dietary measures and sporadic follow-ups characterized the most common adherence pattern among other medical advice.

Table 2 highlights the complexities faced in classifying the ways in which patients attempted to adhere to medical advice. Most patients reported more than one way in which they tried to adhere to medical advice, both simultaneously and over time. For instance, in trying to adhere to medical advice, most patients reported some or all of the ways among making minor adjustments, mixing and changing medicines, and taking breaks or stopping medicines.

Many patients also reported ‘optimal’ adherence in one category and ‘poor’ adherence in another, showing that patients may adhere well to some aspects of their medical advice and not to others. For instance, some patients adhered well to dietary advice but not to medication.

Further, we also encountered some patients who showed shifts from ‘optimal’ adherence to ‘poor’ adherence within each category of medical advice. For instance, patients started with regular tests for their condition but stopped visiting doctors for any follow-ups over time.

## 4. Discussion

In this study, ‘poor’ adherence to medical advice has been examined across three sub-categories- adherence to medication, adherence to lifestyle changes and adherence to medical follow-ups. Within each sub-category, we have discussed varied ways in which patients in our setting have adapted or departed from the advice given to them on the management of NCDs.

### 4.1 A typology of ‘poor’ adherence: What ‘poor’ adherence constitutes in our setting

**Fig 1:**
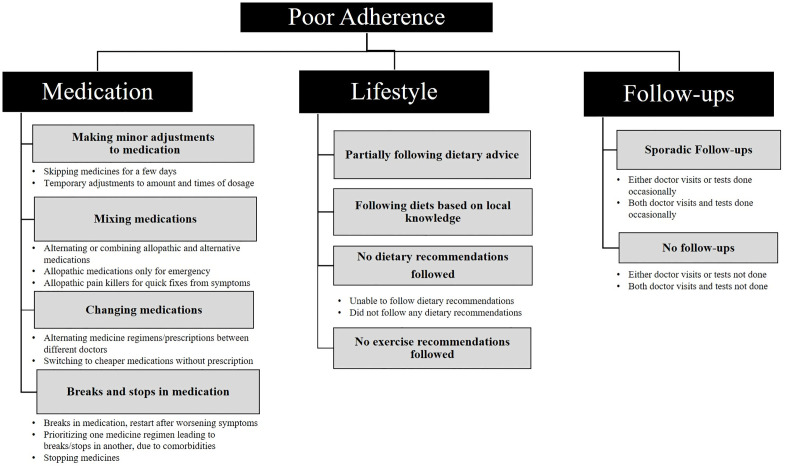
A typology of ‘poor’ adherence: What ‘poor’ adherence constitutes in our setting.

One important contribution of this study is widening the present understanding of ‘poor’ adherence using empirical data from a resource-constrained LMIC setting. Drawing on this data, we have synthesized a typology for ‘poor’ adherence as represented in Fig 1.

This typology adds nuances to some of the existing typologies on adherence to medical advice. To begin with, it reemphasizes the need to shift focus from discussing only adherence to prescribed medication, particularly in the case of NCDs. Most prior studies on poor adherence have focused primarily on adherence to medication [5,6,8,15]. However, in the case of NCDs, adherence to medication alone is not sufficient if not complemented by lifestyle changes and regular monitoring of the disease. Other authors such as Islam et al. have also recognized the need for a broader understanding of adherence that includes medical advice apart from medicines [43]. Accordingly, our typology broadly conceptualizes adherence (or poor adherence) as comprising medication, lifestyle changes, and follow-ups.

Secondly, our typology highlights that ‘poor’ adherence to medical advice in LMICs is a multifaceted concept. We found that patients in our setting engaged in varied practices in their attempts to adhere to medical advice. For instance, ‘poor’ adherence to medication included adjusting dosages, mixing medicines, changing medicines, taking breaks in medicine or stopping medicines. Further, our typology is built from patient practices embedded in contextual specificities, making it more relevant to understanding adherence in vulnerable settings like ours. We found more commonly used typologies in existing literature, such as intentional and unintentional poor adherence [10,11], less helpful in our setting. Intentional and unintentional poor adherence categorizations separate the motivations and choice of patients leading to poor adherence (intentional) from the context and environment of the patient leading to poor adherence (unintentional). However, in our context of urban informal settlements, patients’ choices and ‘intentions’ towards adherence were entwined with the contextual limitations reported by them. For instance, familial support, particularly for male patients, enabled ‘optimal’ dietary adherence, whereas financial constraints led to ‘poor’ adherence to medication. A range of contextual factors- financial conditions, poor quality of public health services, lack of trust in local private doctors, and low education and awareness- shaped adherence to medical advice in our setting.

Third, by mapping patient-wise patterns of adherence based on the typology, we recognize that patients traverse various categories of adherence. This occurs both simultaneously and over time, pointing to the complexity of conceptualizing ‘poor’ adherence in LMIC settings like ours. Unlike existing categorizations of primary and secondary adherence that divide ‘poor’ adherence to medical advice as per the stage of treatment, our typology recognizes diversity in patient-wise adherence patterns over their care-seeking journeys.

In our study setting, it was also challenging to characterize the term ‘medical advice’. First, medical advice received from doctors by patients in our setting was not always comprehensive- some patients reported not being prescribed a diet by their doctors or being provided with only temporary medicines for a few days. In these cases, the question of adhering to medical advice becomes moot since advice is not available to patients in the first place. Further, people obtained informal ‘medical advice’ not only from doctors but from multiple sources such as medicine sellers, neighbors and other patients. Adhering to this broad range of advice negates the very framing of adherence as ‘*concordance of medical advice between doctors and patients*’ [10]. All these complexities point to the need for developing more context-specific definitions of adherence. While existing typologies have been important in moving away from coercive definitions of adherence to being more cognizant of patient concerns [10,11], our study points to the need to be cognizant of their limitations when applied universally. We also seek to problematize the use of the word ‘poor’ in ‘poor adherence’. This generally carries a negative connotation of deviation from prescribed medical advice. Our study shows that ‘poor’ adherence is instead the multiple ways and practices through which patients continually juggle and try to adhere to medical advice, given the enablers and limitations of their context.

Thus, our typology seeks to offer grounded alternatives to conceptualizing ‘poor’ adherence that better explain LMIC contexts. It attempts to broaden the conceptualizations of adherence to medical advice by including medication, lifestyle changes and follow-ups, while nuancing the various ways within each of these categories through which patients attempt to adhere to medical advice. It helps move beyond binary categorizations to recognize the unique adherence journeys of each patient, shaped by context-specific drivers and limitations. Through this it seeks to offer valuable alternatives of qualitative patient-driven categories of ‘poor’ adherence which can help frame interventions in vulnerable contexts.

### 4.2 Framing policy interventions based on the typology

The typology presented through our study can provide crucial inputs for framing better adherence strategies and policy interventions specific to patient needs in urban informal settlements.

Existing interventions are often based on reasons for poor adherence [3,9] or universal categorizations such as intentional and unintentional adherence [8,10]. Given the multifaceted nature of adherence in our context, our typology offers an opportunity to better tailor health strategies according to patient needs.

Our typology shows that strategies for improving adherence cannot approach ‘poor adherence’ as a single uniform category but need to recognize the nuanced and varied ways in which patients attempt to adhere to medical advice. Secondly, our typology stresses the need to tailor interventions based on contextual influences. Third, the typology points to recognizing patients’ unique disease pathways and adherence patterns to provide strategic interventions to improve adherence.

Our typology can be used accordingly to frame community-centric interventions and policy-level changes in improving adherence to medical advice in urban informal settlements. Community-based interventions for improving adherence include providing health promotion messaging and behavior change interventions for patient awareness [44,45]. Community-level interventions through community health workers and peer support groups are one such strategy, which has shown success in LMIC contexts [45–47]. ‘Adherence clubs’ [48] in slum settlements of Nairobi, Kenya, for instance, have benefitted patients by following an integrated approach of providing medicines, health education, group support and timely follow-ups [49]. Our study also points to the need for improving awareness of NCD care among local health providers while enabling them through training and awareness to monitor patients better, provide lifestyle-related advice and ensure medication adherence [50]. The above typology can be useful for both healthcare providers and community health workers in their patient outreach. For instance, patients who take breaks in medicine can be advised on the need to continue them inspite of no symptoms, whereas patients who mix medicines can be made aware of the need to inform doctors of other medicines taken previously/currently, while cautioning patients about taking unprescribed/quick-fix medicines.

Mobile applications [51,52] are also increasingly being used to individualize health messages to improve adherence. MHealth strategies are being adopted by community health workers in LMICs for data collection, patient communication and monitoring towards improving treatment adherence [53,54]. Such applications used by community health workers can utilize our typology as a framework to better map patterns of ‘poor’ adherence among patients and accordingly tailor health messaging.

At the policy level, our typology can help identify systemic changes required for improving adherence. For instance, patients changing or stopping medicines due to financial constraints require health system-related interventions such as training and sensitization of healthcare providers/pharmacies [55], and easy access and availability of affordable medicines [56] at public healthcare services. Similarly, for patients who could not follow diets due to livelihood-related factors, along with awareness, community initiatives such as diet kitchens providing hypertension and diabetes-friendly food can be incentivized [57,58].

Overall, adherence to medical advice can be improved by strengthening public health services [55,56], providing training and support to local health providers, strengthening patient awareness and behavior change through community health workers [44,45] and the use of technology for monitoring [51,52]. Our typology, employed through such strategies, can help identify adherence patterns and accordingly shape improvements. Since our typology is embedded in contextual influences of ‘poor’ adherence, it also helps identify what strategies would be most useful in addressing each category within the typology. For instance, most patients who reported ‘poor’ adherence to diet were due to behavioral factors like lack of awareness or limitations due to workplace and livelihood. Thus, health promotion strategies and community diet kitchens would be a conducive strategy to improve adherence. Comparatively, ‘poor’ adherence to medication was primarily associated with financial constraints, addressing which would require systemic interventions such as improving public health systems. Our study thus serves as an example of how contextualized and patient-centric strategies for improving adherence can be devised based on a grounded typology of adherence. Future research and practice on adherence can use this as a framework to build typologies suited to their context.

### 4.3 Limitations

While the study offers a nuanced typology for ‘poor’ adherence and insights into addressing the issue, it has some limitations. The information gathered from patients about their adherence to medical advice is based on memory recall and could have excluded certain details that patients did not share with us or did not remember due to comorbidities or long patient histories, leading to a recall bias. Although we have diversified the participants, since the patients were sampled through SNEHA’s existing program in the community, the selection of participants included mainly families. We did not have access to recently migrated male workers who were single and living in this area, and we acknowledge that their adherence patterns might have been different. We also acknowledge that our analysis in the present paper is limited to patient narratives, and including healthcare practitioners can help provide more holistic perspectives in future research.

In conclusion, our study shows how existing conceptualizations and categorizations of adherence can be deepened further with more empirical work from LMIC settings. Nuanced understandings of adherence based on grounded research can help frame better policy interventions to improve ‘poor’ adherence to medical advice on NCDs. This study also constructs a framework that can be adapted further in research and practice settings.

## Supporting information

S1 Table ‘Optimal’ Adherence to Medical Advice. (PDF)
