# Supplementary material for: What constitutes ‘poor’ adherence to medical advice for chronic diseases? Insights from a qualitative study among hypertension and diabetes patients in urban informal settlements, Mumbai Metropolitan Region
Source: PLoS One. 2025 Nov 18;20(11):e0324765. doi: 10.1371/journal.pone.0324765 (PMC12626285; doi:10.1371/journal.pone.0324765)
Supplement: S1 Table — (PDF) [file pone.0324765.s001.pdf]

Supporting Information S1 Table

'Optimal' Adherence to Medical Advice

**Optimal Adherence**	**Details**	**Quotes**
Optimal Adherence to Medication	Few patients reported adhering to prescribed medication.<br>Financial stability, support of family and advice from healthcare providers influenced adherence to medication.	"*If it is cough or cold and you don't feel like having medicines is different, but this (hypertension and diabetes) I have to have. I have not stopped it (medicines), because if I stop and I get worse it will not be good. If I forget to take my medicines, my children remind me. They take care of me.*" (Female, 50 years, diagnosed with hypertension and diabetes five years prior to the interaction)
Optimal Adherence to Diet	Some patients mentioned adhering to dietary advice.<br>Awareness about the importance of diet, support from the family and advice from healthcare providers were major reasons for adherence.<br>Gender-based roles in the cultural context of our setting also led women to "take care" of other family members, particularly males in managing diets.	"*My wife helps in diet control. I eat only one spoon of rice, not like earlier times. She does not give me potato. I have two daughters-in-law; she has told them to not give me anything which can harm me. They don't give me.*" (Male, 55 years, diagnosed with diabetes six months prior to the interaction)
Optimal Adherence to Follow-ups	Some patients shared that they regularly visited doctors or did tests after starting their treatment.<br>Such regularity was reported mainly in cases where other family members were also required to visit the doctor/do check-ups or in cases where getting medicines was contingent on visiting doctors/doing tests.	"*Every two to three months I do the checkup at the nearby lab. My husband also has diabetes, so I go with him, and we both do the checkup.*" (Female, 45 years, diagnosed with diabetes 15 years prior to the interaction)
